# Novel ADAM-17 inhibitor ZLDI-8 enhances the in vitro and in vivo chemotherapeutic effects of Sorafenib on hepatocellular carcinoma cells

**DOI:** 10.1038/s41419-018-0804-6

**Published:** 2018-07-03

**Authors:** Yingshi Zhang, Dandan Li, Qiyu Jiang, Shuang Cao, Huiwei Sun, Yantao Chai, Xiaojuan Li, Tianshu Ren, Ruichuang Yang, Fan Feng, Bo-an Li, Qingchun Zhao

**Affiliations:** 1 Department of Pharmacy, General Hospital of Shenyang Military Area Command, Shenyang, 110840 China; 2 Department of Clinical Pharmacy, Shenyang Pharmaceutical University, Shenyang, 110016 China; 3 Research Center For Clinical And Transitional Medicine, The 302nd Hospital of Chinese PLA, Beijing, 100039 China; 4 Hubei Key Laboratory of Novel Chemical Reactor and Green Chemical Technology, Wuhan Institute of Technology, Wuhan, 430073 China; 5 Center for Clinical Laboratory, The 302nd Hospital of Chinese PLA, Beijing, 100039 China

## Abstract

Hepatocellular carcinoma (HCC) is one of the greatest life threats for Chinese people, and the prognosis of this malignancy is poor due to the strong chemotherapy resistance in patients. Notch pathway components mediate cell survival and epithelial–mesenchymal transition (EMT), and also participate in the induction of multi-drug resistance (MDR). In the present study, we demonstrated the discovery of a novel inhibitor for Notch activating/cleaving enzyme ADAM-17, named ZLDI-8; it inhibited the cleavage of NOTCH protein, consequently decreased the expression of pro-survival/anti-apoptosis and EMT related proteins. ZLDI-8 treatment enhanced the susceptibility of HCC cells to a small molecular kinase inhibitor Sorafenib, and chemotherapy agents Etoposide and Paclitaxel. ZLDI-8 treatment enhanced the effect of Sorafenib on inhibiting tumor growth in nude HCC-bearing mice model. These results suggest that ZLDI-8 can be a promising therapeutic agent to enhance Sorafenib’s anti-tumor effect and to overcome the MDR of HCC patients.

## Introduction

Liver diseases represent a medical burden in Asian-pacific region, especially in China^1^. A large proportion of chronic hepatitis finally develop into hepatocellular carcinoma (HCC), an end-stage liver disease (ESLD), even after long-term efficient anti-viral treatment^1–4^. Unfortunately, most HCC patients are first diagnosed at Barcelona Clinic Liver Cancer (BCLC) stage C, the advanced stage which is unsuitable for surgery, and alternative treatments always have poor prognosis or clinical outcome^5–7^. Advanced HCC is also insensitive to cytotoxic chemotherapies^8, 9^. Small molecular protein kinase inhibitor Sorafenib has been demonstrated to significantly improve the survival of advanced HCC patients and benefit in time to progression^10–13^. However, only a low proportion of patients were sensitive to Sorafenib and also associated with gradually increasing drug resistance^14–16^. Therefore, it is urgent to develop novel therapeutic strategies to enhance the efficiency of molecular targeted therapies in HCC treatment.

Notch signaling pathway plays critical role in regulating cell proliferation, differentiation, and cellular injury/stress responses^17, 18^. Recent works have demonstrated that aberrant Notch expression or Notch pathway activation contribute to the development of various malignancies, such as breast cancer, prostate cancer, colorectal cancer, and HCC^19–21^. Upon cell-stress, e.g., ionizing radiation or cytotoxic chemotherapeutic agents, Notch will be activated and cleaved by metalloproteases domain-17 (ADAM-17), leading to the release of the Notch intracellular domain (NICD)^22–24^. Then, NICD translocates into nucleus and mediates the transcription of Notch’s targeted genes, such as Bcl-2, Survivin or IAPs^22–24^. Inhibition of Notch pathway’s activation is a promising strategy to increase anticancer effects of antitumor approaches^25^. Yang et al. and Gyöngyösi et al.^26, 27^ provided the clues that Notch-1 signaling affects the effect of Sorafenib. Jia et al. and Kang et al.^28, 29^ reported that Rhamnetin, a polyphenol structure containing flavonoid compound extracted from Hippophae rhamnoides Linn, enhanced the sensitivity of HCC or NSCLC cells to ionizing radiation (IR) and chemotherapies by inhibiting Notch pathway. Therefore, development of Notch pathway’s inhibitor is a promising strategy to enhance the efficacy of antitumor agents on HCC cells.

In the present work, we describe the discovery of novel ADAM-17 inhibitor ZLDI-8 (previously named as IAC-8 or inhibitor of ADAM-17 compound No. 8) [5-((1-(2-(2,4-dimethylphenoxy) ethyl) -2-methyl-1H-indol-3-yl) methylene) -2-thioxodihydropyrimidine-4,6 (1H,5H) -dione] (Suppl Fig. 1), by using virtual molecular docking^30^. Treatment of ZLDI-8 significantly disrupted the activity of Notch pathway in HCC cells and inhibited the epithelial–mesenchymal transition (EMT) process of HCC cells. Moreover, ZLDI-8 treatment enhanced the susceptibility of HCC cells to Sorafenib, Etoposide, and Paclitaxel. ZLDI-8 treatment also enhanced the effect of Sorafenib on inhibiting in vivo HCC tumor.

## Materials and methods

### Agents and cell culture

ZLDI-8 (Cat. No.: AO-299/41409126) was purchased from Specs Corporation, Zoetermeer, Netherlands. Anti-tumor agents, Sorafenib (Cat. No.: S7397), Paclitaxel (Cat. No.: S1150), and Etoposide (Cat. No.: S1225) were purchased from Selleck Corporation, Houston, Texas, USA. Hepatic cell lines, HepG2 (a HCC cell line) or MHCC97-H (a highly aggressive HCC cell line), were cultured under recommended culture conditions described in our previous publications^31, 32^. LM-3 (HCC-LM3), a highly aggressive HCC cell line, was a kind gift from Prof. Shoujun Yuan in Department of Pharmacology and Toxicology, Beijing Institute of Radiation Medicine, 100081 Beijing, China. LM-3 is cultured in DMEM adding 10% FBS under 37 ℃ with 5% CO_2_. For survival inhibition analysis, cells were treated with indicated concentration of agents, as shown in Supplementay Table 1. Next, the cells were MTT analyzed and the absorbance was measured using a multifunctional microplate-reader at 490 nm. The inhibition rate of antitumor agents was calculated as (O.D. 490 control group−O.D. 490 administration group)/(O.D. 490 control group−O.D. 490 blank group) × 100%). And the relative survival cell number was calculated as 100%−inhibition rate. Assays were performed three independent times with similar results.

### Molecular docking

To explore the binding mode of ZLDI-8 (AO299/41409126) with ADAM-17^33^, molecular docking simulation studies were carried out by using the SURFLEX-DOCK module of the SYBYL 6.9 package version (Tripos International, St. Louis, MO, USA). X-ray crystal structure of ADAM-17 (PDB ID code: 2DDF) was obtained from the Protein Data Bank (PDB) (http://www.wwpdb.org). Ligands and water molecules were removed from the crystal structures of the protein, and hydrogen atoms were added. According to the central role of Zinc ions in docking, it was retained in the protein structure.

### Western blot analysis

The antibody Cat. No.: sc-373891) against Notch NICD was purchased from Santa Cruz Corporation, Dallas, Texas, USA. Antibodies against Survivin (Cat. No.: ab76424)), c-IAP-2 (Cat. No.: ab25939), c-IAP-1 (Cat. No.: ab108361), Lamin A/C (Cat. No.: ab169532), β-Actin (Cat. No.: ab8226), GAPDH (Cat. No.: ab8245), Ki67 (Cat. No.: ab16667), PARP (Cat. No.: ab74290), cleaved PARP (Cat. No.: ab219953), and Anti-rabbit IgG (Cat. No.: ab6728) and anti-mouse IgG (Cat. No.: ab190475) antibodies conjugated with horseradish peroxidase (HRP) were purchased from Abcam cooperation (Cambridge, UK). Total protein samples were extracted from HCC cells and performed by SDS–PAGE, and transprinted to poly-vinylidene fluoride (PVDF) membranes (Millipore, Billerica, MA, USA). The membranes were blocked and then incubated with primary antibodies. The blots were then incubated with the HRP-conjugated secondary antibodies. At last, blots were developed with enhanced chemiluminescence reagents (Pierce, Rockford, IL, USA) by X-ray films.

### Transwell analysis

MHCC-97H cell were treated with indicated concentrations of anti-tumor agents and analyzed by transwell assays performed in 24-well plates chamber (Cat. No.: Costar 3422, Corning, Lowell, MA, USA) fitted with a polyethylene terephthalate filter membrane with 8-μm pores. The invasion-transwell or migration-transwell was performed following the methods described by Ji et al. and Liang et al.^34, 35^.

### Flow cytometer

For apoptosis analysis, cells were labeled with FITC-Annexin V and 7-AAD followed manufacturer’s instructions (Cat. No.: 556547, BD Biosciences, Franklin Lakes, NJ, USA)^36^. For cell-cycle analysis, cells were labeled with PI according to manufacturer’s instructions (Cat. No.: 550825, BD Biosciences, Franklin Lakes, NJ, USA). Then, cells were detected by the FACScalibur Flow Cytometer (Becton Dickinson, BD Biosciences, Franklin Lakes, NJ, USA).

### Animal experiments

All the animal experiments were reviewed and approved by the Institutional Animal Care and Use Committee of Shenyang Pharmaceutical University. To produce the subcutaneous tumor model^37, 38^, MHCC-97H or LM-3 cells were injected into nude mice (1 × 10^6^ cells per animal). After 2–3 days growth, animals received 2 mg/kg, 1 mg/kg, 500 μg/kg, or 200 μg/kg concentration of ZLDI-8 administrated i.p. or 2.5 mg/kg Sorafenib administrated i.g. every 2 day for 20 days. Tumors were harvested and their volumes and weights were measured.

To produce an intrahepatic tumor model^39, 40^, MHCC-97H cells were seeded to produce subcutaneous tumors. Tissues (about 1 mm^3^ in volume) were directly inoculated into the right lobe of the liver. After 2–3 days growth, animals received 500 μg/kg ZLDI-8 administrated i.p. or 2.5 mg/kg Sorafenib administrated i.g. every 2 day for 20 days. Nude mice were injected intravenously with 200 μCi of ^18^F radio-labeled fluorodeoxyglucose (^18^F-FDG), and the animals were examined using a micro-positron emission tomography (^Micro^PET) scanner (Philips Corporation, Amsterdam, Holland)^41, 42^. CT scan for 2 min and PET for 10 min were performed after 30 min of the FDG injection. A NaI (Tl) well counter (China Atom Corp., Beijing, China) was used to measure the radioactivity of liver compared with blood^41, 42^.

To produce an in vivo metastatic HCC model to mimic advanced HCC^43^, MHCC-97H or LM-3 cells were injected into nude mice’s liver via hepatic portal vein injection. After 2–3 days growth, animals received 500 μg/kg ZLDI-8 administrated i.p. or 2.5 mg/kg Sorafenib administrated i.g. every 2 day for 20 days. Then, nude mice were injected intravenously with 200 μCi of ^18^F radio-labeled fluorodeoxyglucose (^18^F-FDG), and the animals were examined using a micro-positron emission tomography (^Micro^PET) scanner (Philips Corporation, Amsterdam, Holland). CT scan for 2  min and PET for 10  min were performed after 30 min of the FDG injection. A NaI (Tl) well counter (China Atom Corp., Beijing, China) was used to measure the radioactivity of liver compared with blood. Then, liver organs photographs were analyzed by an Image J Software (Version No.: 1.51j8, National Institutes of Health, Bethesda, Maryland, USA)^41, 42^. The percentage of nodule’s areas was calculated by following the indicated methods provided by Xie et al.^43^, and indicated the relative HCC amount. Masson staining kits were purchased from Zhan-shan-jin-qiao Corporation, Beijing, China. The Masson staining of tissues was performed following the instruction provided by the manufacturer.

### Statistical analysis

The *IC*_*50*_ values of anti-tumor agents on HCC cells were calculated by Origin 6.1 software. All statistical significance analyses were performed using SPSS 19.0 statistical software. A two-tailed value of *p* < 0.05 was considered to be statistically significant. All group comparisons were annlyzed by one-way ANOVA with or without post-hoc multiple comparisons by Bonferroni.

## Results

### ZLDI-8 inhibits the activity of Notch signaling pathway

**Fig. 1:**
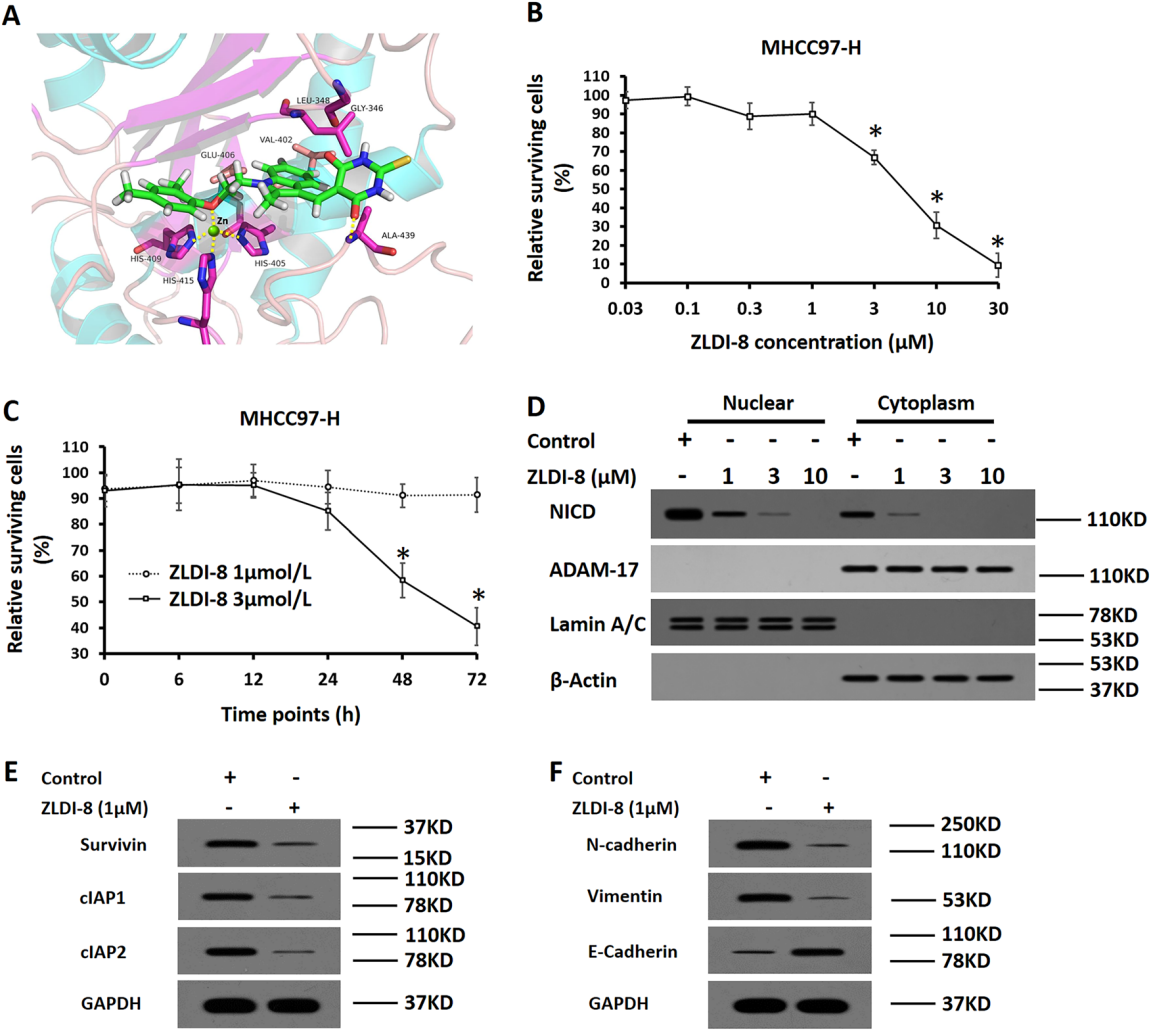
Identification of the non-cytotoxic concentration of ZLDI-8 in MHCC97-H cells. **a** Molecular docking for the interaction between ZLDI-8 and ADAM-17. **b** MHCC97-H cells were treated with indicated concentration of ZLDI-8, and the inhibition rates were calculated. **c** The effect of 1 μmol/L or 3 μmol/L ZLDI-8 was examined at each time point. **d** MHCC97-H, which were treated with indicated concentration of ZLDI-8, were fractionated into cytoplasmic and nuclear fractions. The fractions were detected with anti-NICD antibody and ADAM-17 antibodies. β-actin was chosen as a cytoplasmic marker and Lamin A/C was the nuclear indicator. **e**, **f** MHCC97-H cells, which were treated with 1 μmol/L ZLDI-8, were examined to detect the protein level of anti-apoptosis/pro-survival (**e**) or EMT (**f**) related regulators

First, the 3D structure of ZLDI-8 and ADAM-17 interaction was predicted by molecular docking software (Fig. 1a). In this model, the indole of compound ZLDI-8 embedded deeply into the cavity and paralleled with the α-helix from Leu395 to Gly412. Oxygen atom in the phenol ether structure of compound ZLDI-8 forms a tetrahedral structure with the residues ^409^His, ^415^His, and ^405^His. The Zinc ions maintains the stability of the tetrahedron coordination bond (yellow); and the oxygen atom in thioxodihydropyrimidine structure forms a hydrogen bond (orange) with residue ^439^Ala.

**Fig. 2:**
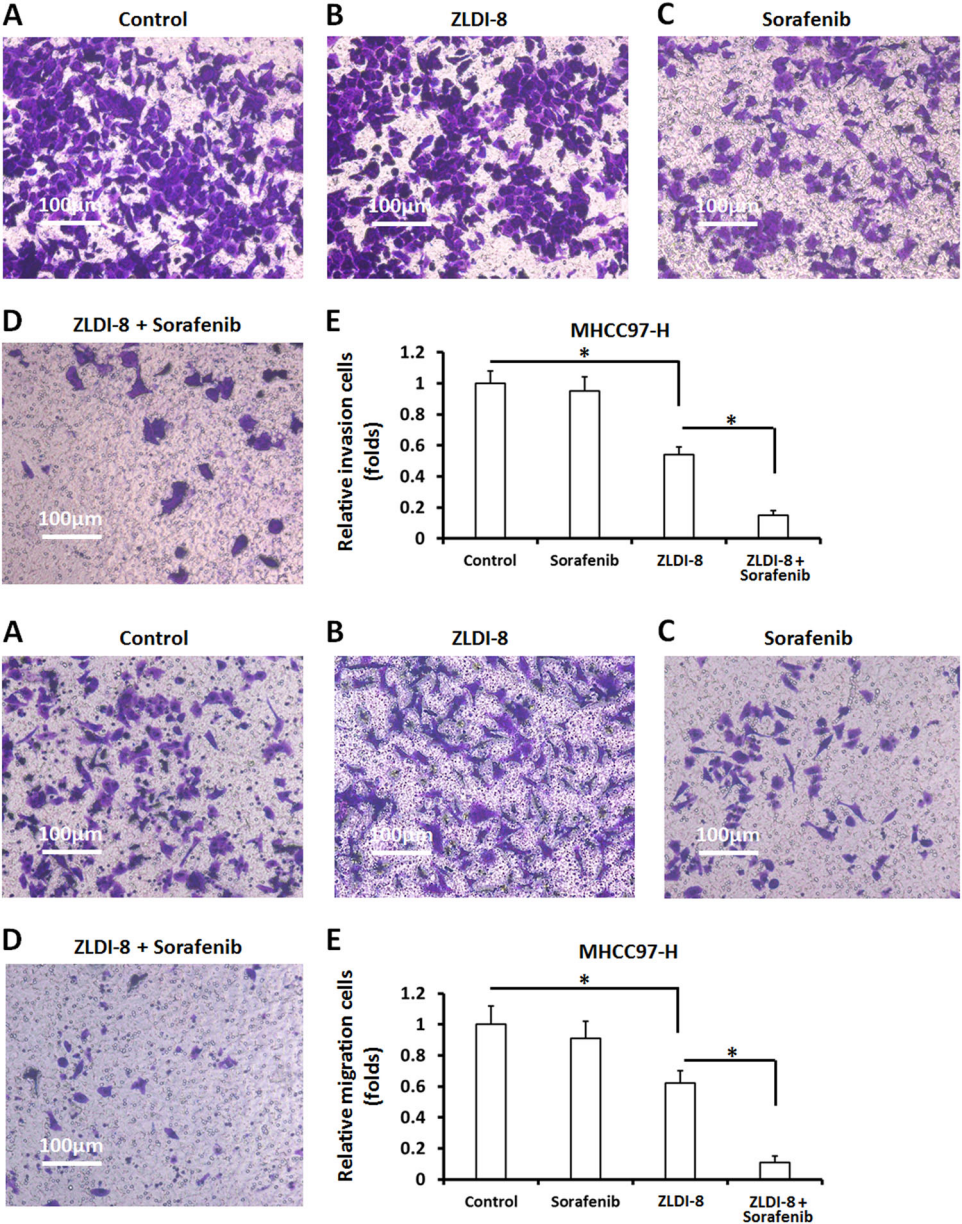
Pre-treatment of ZLDI-8 enhances the inhibitory activation of Sorafenib on MHCC97-H cells’ in vitro invasion and migration. MHCC97-H cells, which were pretreated with 1 μmol/L ZLDI-8 or solvent control (1‰ DMSO), were treated with the *IC*_*50*_ concentration of Sorafenib. Cells were then harvested and analyzed for trans-well assays. The invasion **a** and migration **b** of MHCC97-H cells were shown as the represented photographs or the relative invasion/migration cell numbers. *p < 0.05 vs. Sorafenib or control; *p < 0.05 vs. ZLDI-8 or control

Next, we tested the cytotoxic kinetics of ZLDI-8. MHCC97-H cells were treated with indicated concentrations (Fig. 1b) of ZLDI-8 at different time points (Fig. 2c). Our data showed that *IC*_*50*_ value of ZLDI-8 is 5.32 ± 0.46 μmol/L: it emerges cytotoxic effect on MHCC97-H cells at 3 μmol/L, 10 μmol/L, and 30 μmol/L but not at 1 μmol/L (Fig. 1b, c).

Then, the effect of ZLDI-8 on Notch signaling pathway was determined. As shown in Fig. 1d, at non-cytotoxic concentration 1 μmol/L, ZLDI-8 significantly decreased the level of NICD and the accumulation of NICD in the nucleus. Moreover, ZLDI-8 could also reduce the expression of pro-survival/anti-apoptosis regulators, Survivin and cIAP1/2 (known as cellular inhibitor of apoptosis 1/2), two downstream proteins in Notch pathway. ZLDI-8 treatment also increased the expression of epithelial marker E-Cadherin and reduced mesenchymal markers N-Cadherin and Vimentin (Fig. 1e, f). We thereby selected 1 μmol/L as the preferred concentration in our following studies to demonstrate the direct effect of ADAM-17 or Notch signaling blockage on HCC cell growth and drug resistance, without the interference of chemical toxicity per se on cells.

### ZLDI-8 enhances Sorafenib-mediated impairment of HCC cell survival

We further tested whether Notch signaling blockage by ZLDI-8 can also facilitate Sorafenib’s effect. As predicted, pre-treatment of ZLDI-8 at 1 μmol/L enhanced the activity of Sorafenib on HCC cells: upon co-administration, the *IC*_*50*_ values of Sorafenib decreased from 2.62 ± 0.29 μmol/L to 0.30 ± 0.11 μmol/L in MHCC97-H and 1.13 ± 0.05 μmol/L to 0.15 ± 0.01 μmol/L in HepG2, respectively (Tables 1 and 2).

**Table 1:** ZLDI-8 enhanced the sensitivity of MHCC97-H cells to Sorafenib, Etoposide or Paclitaxel

Compounds	Sorafenib	Etoposide	Paclitaxel
	*IC*_*50*_ Value (μmol/L)		
Solvent control	2.62 ± 0.29	0.11 ± 0.01	0.14 ± 0.02
ZLDI-8	0.30 ± 0.11	0.06 ± 0.01	0.05 ± 0.00

**Table 2:** ZLDI-8 enhanced the sensitivity of HepG2 cells to Sorafenib, Etoposide or Paclitaxel

Compounds	Sorafenib	Etoposide	Paclitaxel
	*IC*_*50*_ Value (μmol/L)		
**Solvent control**	1.13 ± 0.05	0.34 ± 0.03	0.13 ± 0.01
**ZLDI-8**	0.15 ± 0.01	0.11 ± 0.01	0.02 ± 0.00

By the transwell assay, we then found that ZLDI-8 treatment alone did not significantly inhibited MHCC97-H cell invasion (Fig. 2a) and migration (Fig. 2b). However, when ZLDI-8 was co-administrated with Sorafenib, it further facilitated the antitumor effects of Sorafenib (Fig. 2a, b), suggesting that ZLDI-8 per se did not significantly inhibit the invasion or migration of MHCC97-H, but can increase the susceptibility of tumor cells, HCC cells in this case, to Sorafenib.

**Fig. 3:**
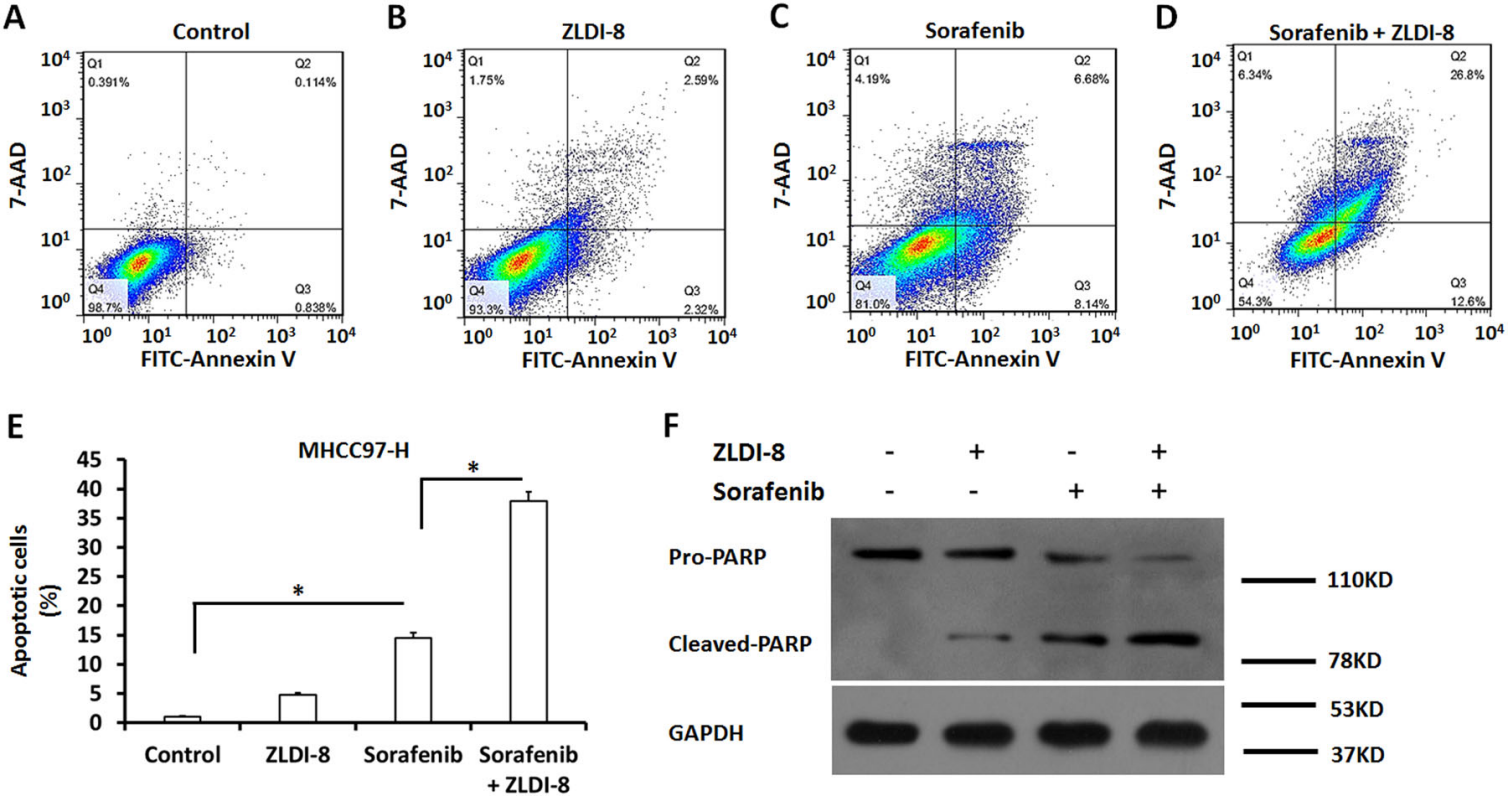
Pre-treatment of ZLDI-8 enhances the efficiency of Sorafenib inducing MHCC97-H cells’ apoptosis. MHCC97-H cells, which were pretreated with 1 μmol/L ZLDI-8 or solvent control (1‰ DMSO), were treated with the *IC*_*50*_ concentration of Sorafenib. Cells were then harvested and analyzed for flow cytometer. The apoptosis of MHCC97-H cells were shown as the represented photographs or the apoptotic cell percentages. *p < 0.05 vs. Sorafenib or DMSO; *p < 0.05 vs. ZLDI-8 or DMSO

Moreover, Sorafenib induced apoptosis of MHCC97-H cells from 1.02 to 14.48% (Fig. 3a, c). ZLDI-8 alone did not significantly induced the apoptosis of MHCC97-H cells. Pre-treatment of ZLDI-8 further increased the MHCC97-H cell apoptosis under Sorafenib administration from 14.48 to 37.92% (Fig. 3a, c and d). In consistent to this, treatment of ZLDI-8 enhanced the cleaving of PARP protein induced by Sorafenib (Fig. 3f). Thus, our data suggest that ZLDI-8 enhances the in vitro antitumor effect of Sorafenib on HCC cells.

### ZLDI-8 enhances in vivo anti-tumor effect of Sorafenib on HCC cells

**Fig. 4:**
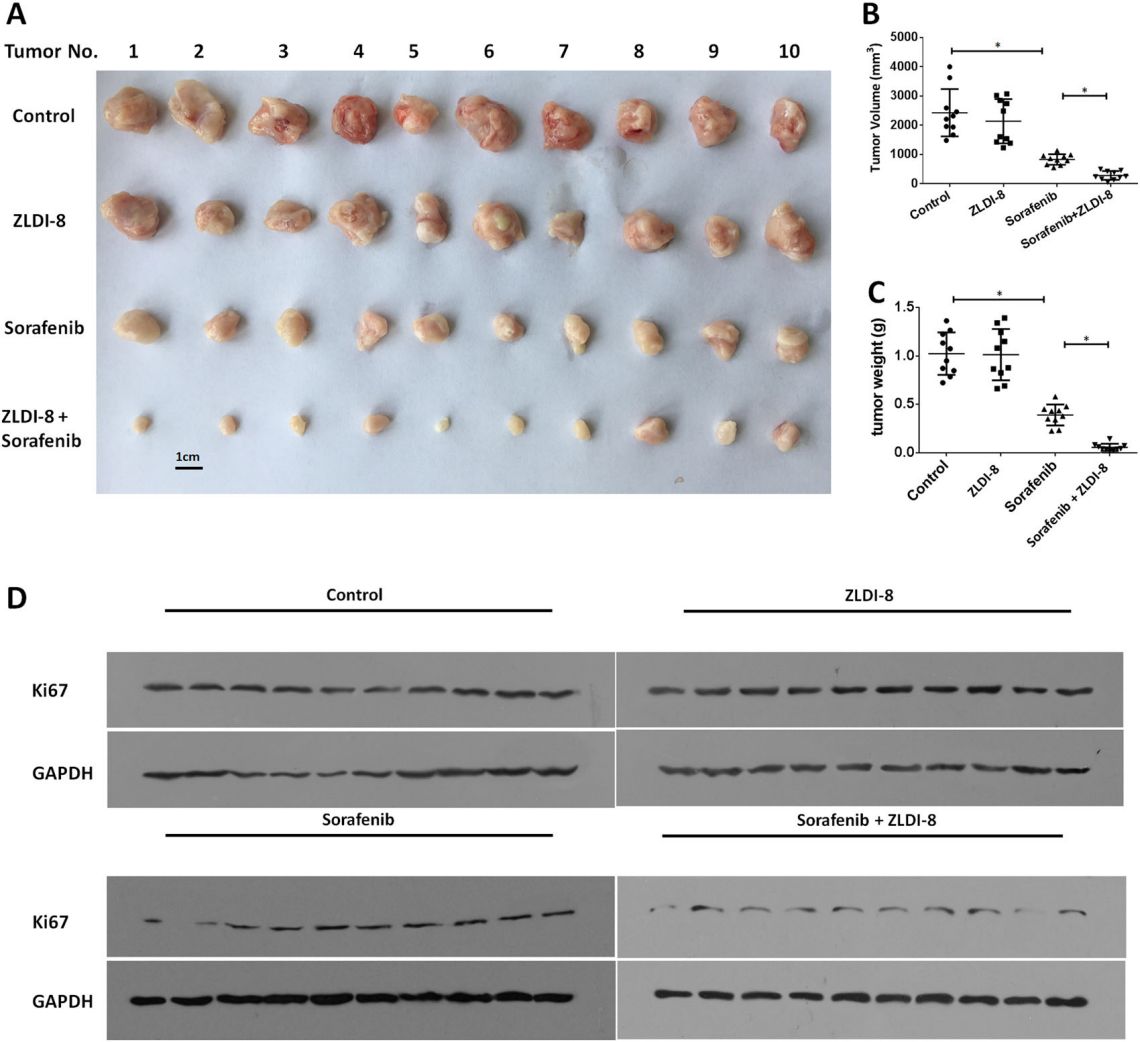
ZLDI-8 increases in vivo antitumor effect of Sorafenib on MHCC97-H cells’ subcutaneous growth. MHCC97-H cells were seeded into nude mice to form subcutaneous tumors. **a**–**c** The anti-tumor activity of ZLDI-8 and Sorafenib were measured in xenograft nude mice. The results were shown as photographs (**a**), tumor volumes (**b**), or tumor weight (**c**). **d** The expression of a proliferation marker, Ki67, was identified by western blot experiments. *p < 0.05 vs. Sorafenib or control; *p < 0.05 vs. ZLDI-8 or control

**Fig. 5:**
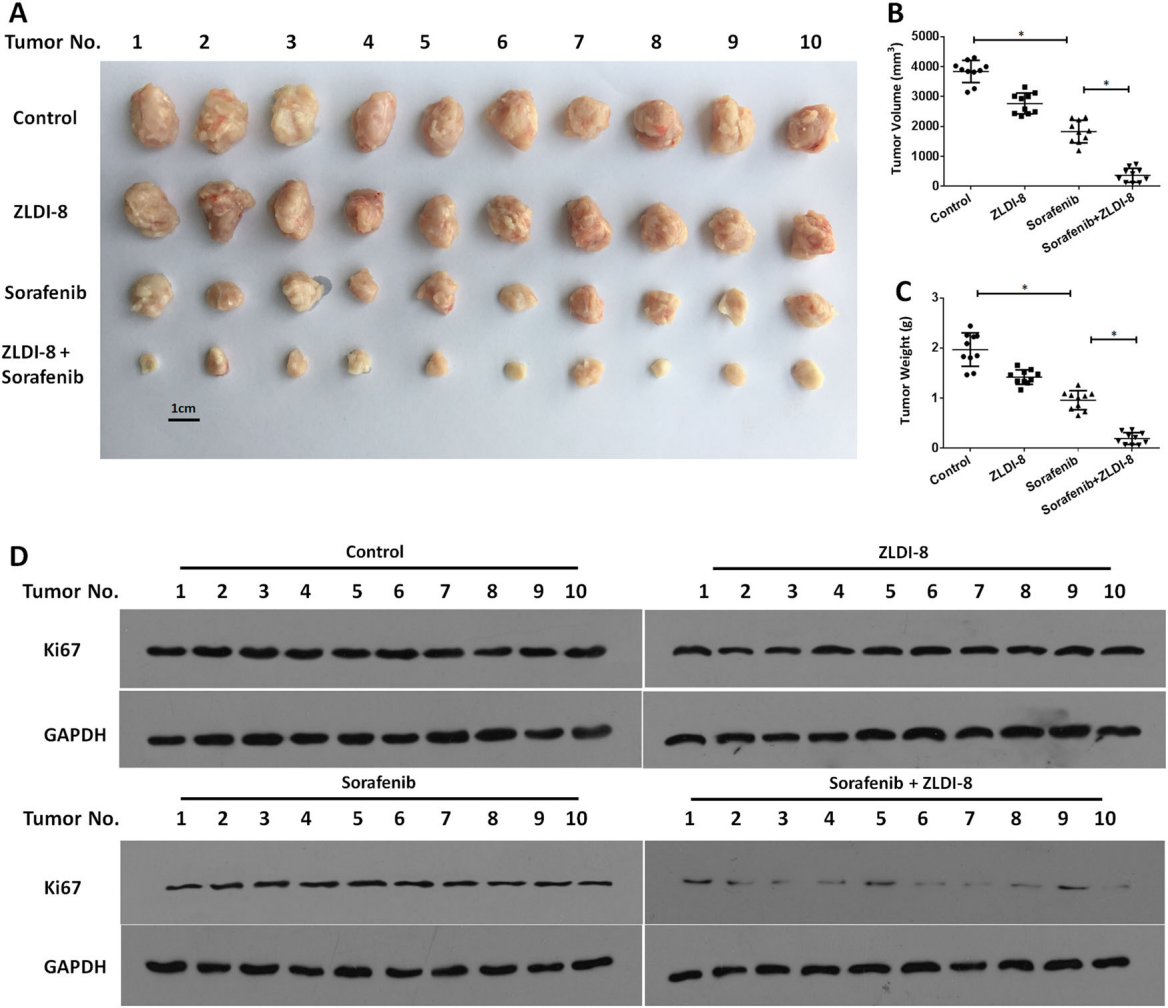
ZLDI-8 increases in vivo antitumor effect of Sorafenib on LM-3 cells’ subcutaneous growth. LM-3 cells were seeded into nude mice to form subcutaneous tumors. **a**–**c** The anti-tumor activity of ZLDI-8 and Sorafenib were measured in xenograft nude mice. The results were shown as photographs (**a**), tumor volumes (**b**) or tumor weight (**c**). **d** The expression of a proliferation marker, Ki67, was identified by western blot experiments. *p < 0.05 vs. Sorafenib or control; *p < 0.05 vs. ZLDI-8 or control

Next, we focused the effect of ZLDI-8 on in vivo subcutaneous tumor growth model. Tumor bearing mice were treated with vehicle solution (the solvent control), Sorafenib, ZLDI-8 or Sorafenib+ZLDI-8 and the tumor growth was monitored by measuring tumor volume and weight. In consistent to our in vitro findings, 500 μg/kg ZLDI-8 did not inhibit the subcutaneous growth of MHCC97H cells but maintained the inhibitory ability on Notch pathway (Suppl Figs. 2 and 3), whereas Sorafenib+ZLDI-8 treatment had greater tumor restriction than Sorafenib treatment (Fig. 4a–c). In addition, similar results were obtained in the in vivo tumor growth of LM-3, another highly aggressive HCC cell line (Fig. 5). As Ki67 inhibition is one of the therapeutic effects of Sorafenib, we detected Ki67 in the tumor cells and found significant reduction in Sorafenib+ZLDI-8 treatment groups (Figs. 4 and 5).

**Fig. 6:**
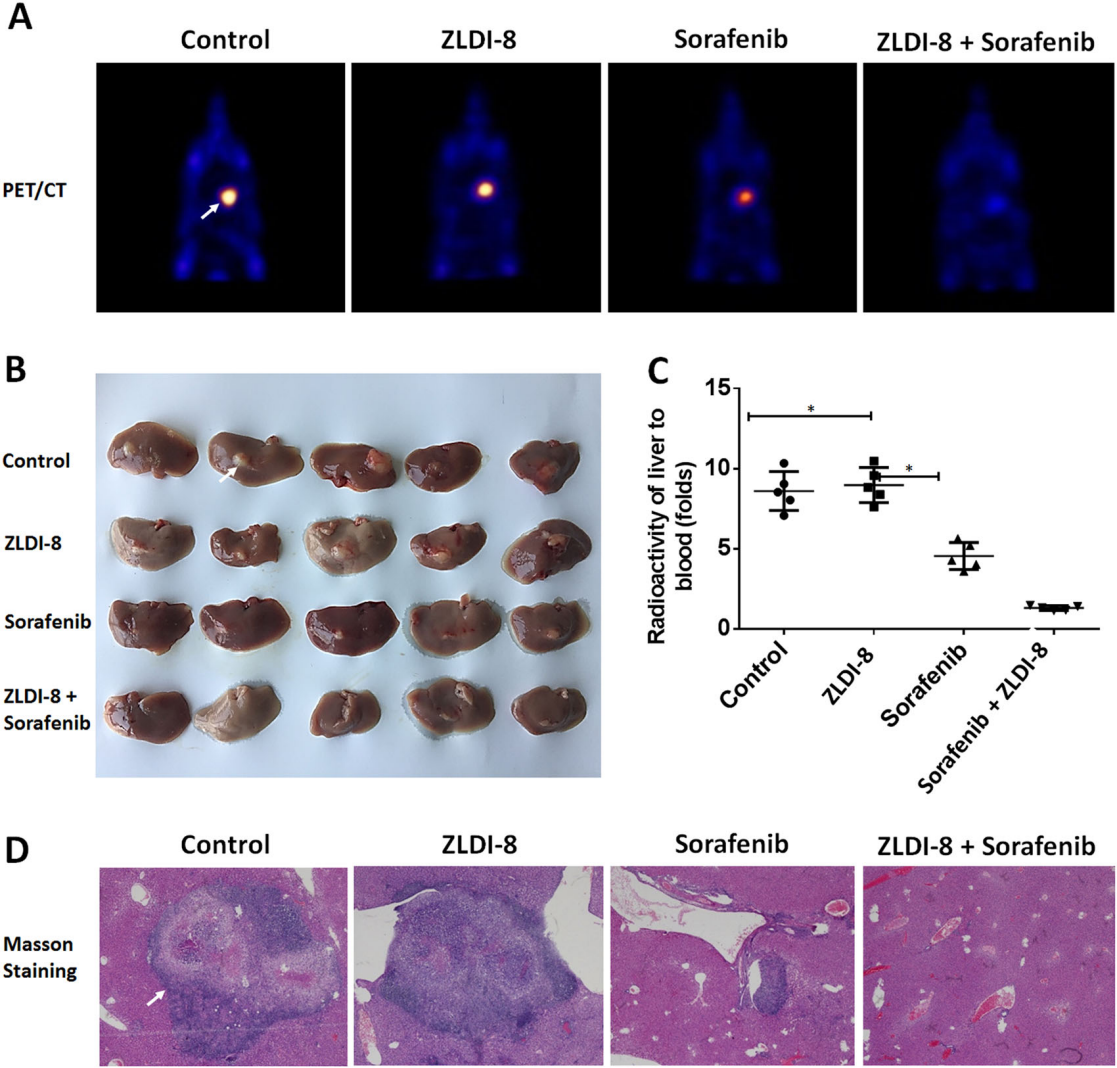
ZLDI-8 increases in vivo sensitization of Sorafenib in an intra-hepatic tumor model. MHCC97-H cells were injected into the right lobe of the liver. Animals were received solvent control, ZLDI-8, Sorafenib or ZLDI-8+Sorafenib. Next, ^18^F-FDG/PET images (*n* = 5) were obtained (**a**) and the results of the PET were confirmed by the radioactivity of ablated livers (**b**). The PET imagines of ablated livers (**b**) and Masson staining (**c**) were also showed. The arrows indicate intrahepatic tumor nodules. *p < 0.05 vs. Sorafenib or control; *p < 0.05 vs. ZLDI-8 or control

Although the subcutaneous tumor is a in vivo tumor model, it could not satisfactorily mimic the intrahepatic growth of MHCC97-H cells. Therefore, the effect of ZLDI-8+Sorafenib treatment was examined in intra-hepatic/in situ liver tumor model, in which PET imaging an Masson staining used to indicated the in situ tumor growth in intra-hepatic nodules region. Sorafenib clearly decreased the nodules formed by MHCC97-H in liver; whereas, ZLDI-8 alone did not significantly affect the intrahepatic growth of those cells. ZLDI-8+Sorafenib treatment had significantly enhanced the anti-tumor effect comparing to Sorafenib treatment alone (Fig. 6a–c). The in vivo data suggests that ZLDI-8 enhances the in vivo antitumor capacity of Sorafenib and functions as sensitizer of Sorafenib in HCC treatment.

### ZLDI-8 enhances the anti-tumor effect of Sorafenib on HCC cells’ in vivo metastasis

**Fig. 7:**
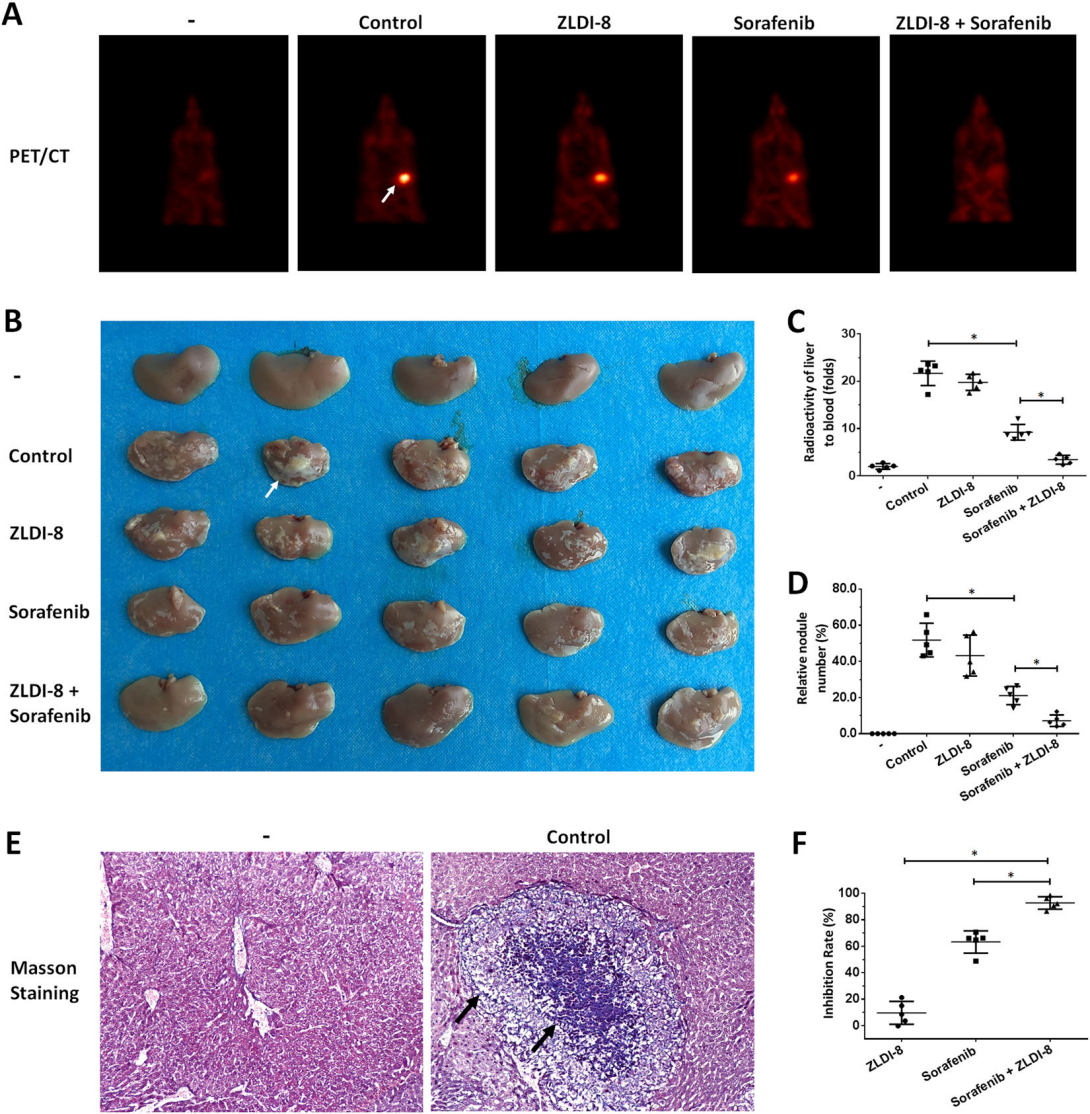
ZLDI-8 enhanced the antitumor effect of Sorafenib on in vivo metastasis of MHCC97-H cells. Single cells of MHCC97-H was injected into nude mice’s liver *via* hepatic portal vein. Animals were received solvent control, ZLDI-8, Sorafenib or ZLDI-8 + Sorafenib. Next, ^18^F-FDG/PET images (*n* = 5) were obtained (**a**) and the results of the PET were confirmed by the imagines (**b**) or radioactivity of ablated livers (**c**). **d** The relative nodules number was shown as quantitative results. Nodules in liver organ were confirmed by the Masson staining results (**e**). The inhibition rate of ZLDI-8, Sorafenib or ZLDI-8 + Sorafenib was calculated from radioactivation and shown (**f**). The PET (**b**) and Masson staining (**c**) were also showed. The arrows indicate intrahepatic tumor nodules. *p < 0.05 vs. Sorafenib or control; *p < 0.05 vs. ZLDI-8 or control

**Fig. 8:**
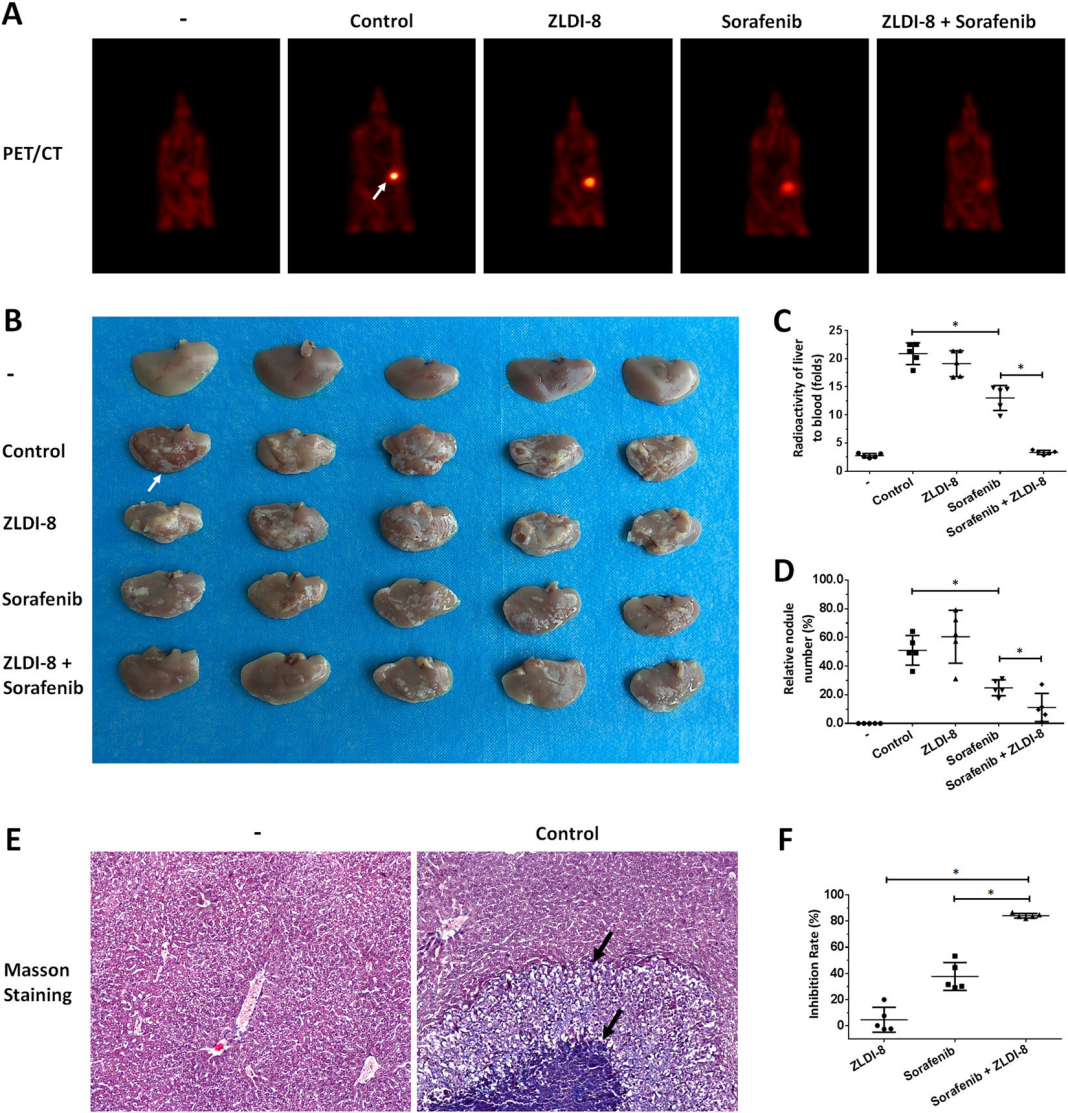
ZLDI-8 enhanced the antitumor effect of Sorafenib on in vivo metastasis of LM-3 cells. Single cells of LM-3 was injected into nude mice’s liver via hepatic portal vein. Animals were received solvent control, ZLDI-8, Sorafenib or ZLDI-8+Sorafenib. Next, ^18^F-FDG/PET images (*n* = 5) were obtained (**a**) and the results of the PET were confirmed by the imagines (**b**) or radioactivity of ablated livers (**c**). **d** The relative nodules number was shown as quantitative results. Nodules in liver organ were confirmed by the Masson staining results (**e**). The inhibition rate of ZLDI-8, Sorafenib or ZLDI-8+Sorafenib was calculated from radioactivation and shown (**f**). The PET (**b**) and Masson staining (**c**) were also showed. The arrows indicate intrahepatic tumor nodules. *p < 0.05 vs. Sorafenib or control; *p < 0.05 vs. ZLDI-8 or control

Next we studied the effect of ZLDI-8 and Sorafenib combination on HCC in vivo metastasis. After hepatic portal vein injection, MHCC97-H cells formed multiple and diffuse nodules in nude mice’s liver (Fig. 7). Upon treatment, we found ZLDI-8 significantly enhanced the effect of Sorafenib on decreasing nodule formation. The results are shown as PET screening images (Fig. 7a), photographs of liver organs (Fig. 7b) radio-activation of liver organs (Fig. 7c) or the relative area of nodules (Fig. 7d). Nodules are confirmed by Masson staining (Fig. 7e) and the inhibition rate of ZLDI-8, Sorafenib or Sorafenib+ZLDI-8 is shown in Fig. 7f. Similar results were obtained in LM-3, another highly aggressive HCC cell line (Fig. 8). Therefore, ZLDI-8 could enhanced the anti-tumor effect of Sorafenib on HCC in vivo metastasis.

### ZLDI-8 enhances the anti-tumor activity of traditional cytotoxic-agents

**Fig. 9:**
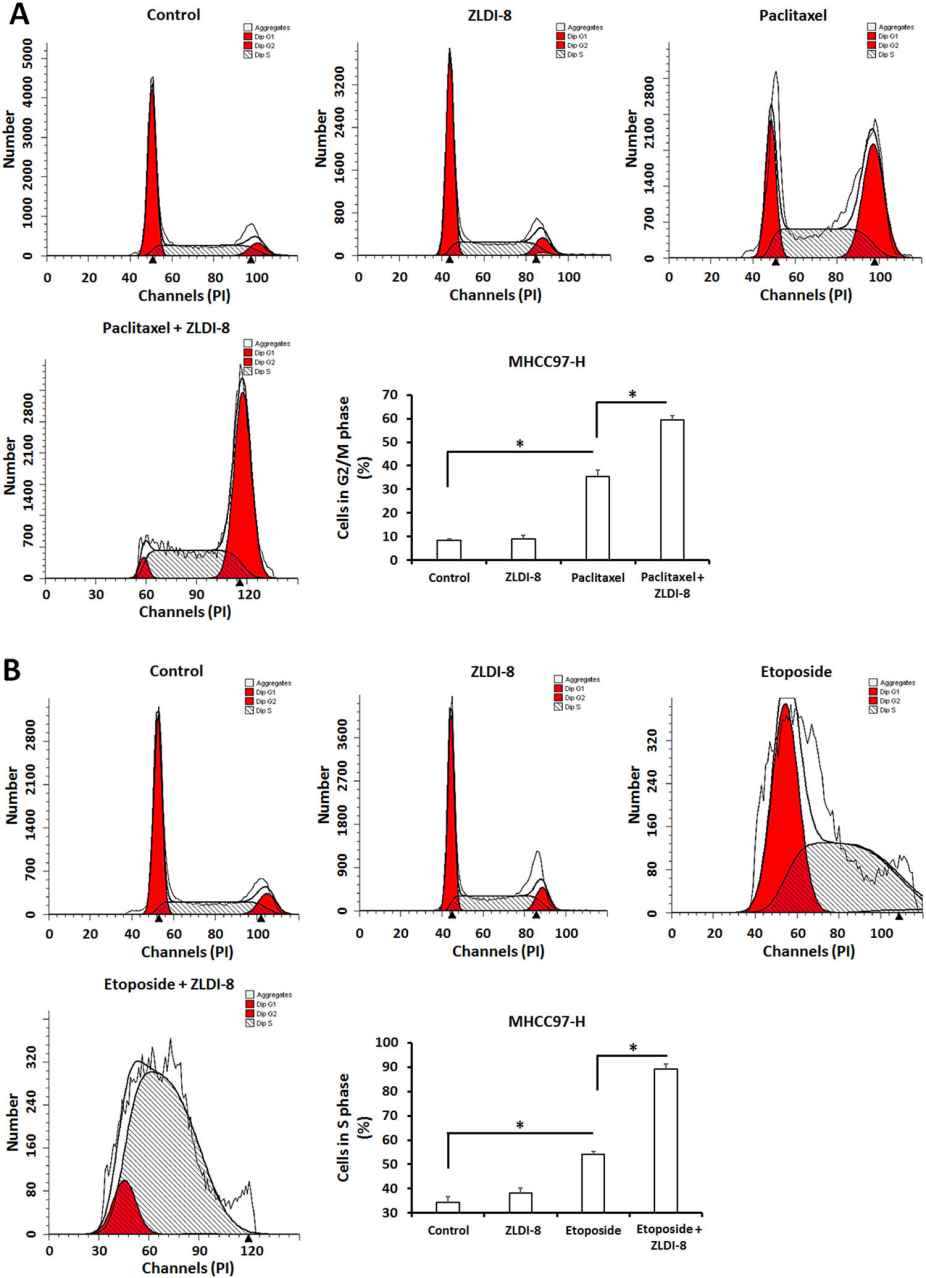
Effect of ZLDI-8 on cytotoxic activity of Etoposide or paclitaxel on MHCC97-H cell-cycle arrest. Cells, were pre-treated with ZLDI-8, were treated with *IC*_*50*_ concentration of Etoposide (**a**) or Paclitaxel (**b**). Then, the cells were harvested and flow cytometer was performed. Results were shown as photographs or mean ± SD. *p < 0.05 vs. Sorafenib or control; *p < 0.05 vs. ZLDI-8 or control

Next, we aimed to study whether ZLDI-8 treatment can increase tumor cell’s susceptibility to traditional cytotoxic-chemotherapeutic agents, such as Etoposide or Paclitaxel. As our data showed, ZLDI-8 significantly increased the inhibitory capacities of Etoposide or Paclitaxel on HCC cell survival: the *IC*_*50*_ values of Etoposide or Paclitaxel on HCC cells were correspondingly decreased (Tables 1 and 2). We further measured the effect of ZLDI-8 on Etoposide or Paclitaxel induced HCC cell-cycle arrest. With ZLDI-8 pre-treatment, the rate of Paclitaxel-induced G2/M-phase arrest increased from 35.37 to 59.37% (Fig. 9a); With ZLDI-8 pre-treatment, the rate of Etoposide-induced S-phase arrest increased from 53.99 to 89.27% (Fig. 9b). Therefore, our data demonstrate that ZLDI-8 enhances chemotherapy effects on tumor cell proliferation blockage, induction of apoptosis and cell-cycle arrest by inhibiting Notch pathway and blocking chemical resistance.

## Discussion

During the past few years, despite improvement in early screening and diagnosis of HCC, most patients still were diagnosed with advanced HCC, with limited options in clinical treatment^44–46^. Currently, there is no effective systemic chemotherapy for advanced stage HCC, and its multi-drug resistance (MDR) remains as a major obstacle for novel approach discovery^45–47^. Moreover, the radioresistance of HCC is also a critical obstacle^48, 49^. Clinical investigations have reported that the one-third, two-third, or whole liver can only be safely irradiated with 90, 47, or 31 Gy does of ionizing radiation (IR), respectively; however, these doses do not reach the required volume of HCC-controling dose^49–51^. Sorafenib is the first approved front-line anti-tumor agents for advanced HCC^11, 12, 52^. Recently, some other molecular target agents, e.g., Regorafenib or apatinib, were also approved for advanced HCC treatment^53, 54^. Although these molecular targeted agents bring new hope for patients with advanced HCC, the efficacy of these agents is still far from satisfying. To aim to solve this problem, our current work provides new light in advanced HCC’s treatment (Supple Fig. 4). Inhibition of Notch pathway via ZLDI-8 reduces the expression of or pro-survival and EMT related genes. Since we want to develop a promising agent to enhance the sensitivity of HCC cells to anti-tumor compounds, the dose of ZLDI-8 used in this work should not show significantly cytotoxic activities of ZLDI-8 itself. Even though the MHCC97-H cell survival was not affected in by non-cytotoxic dose (1 μmol/L) of ZLDI-8 compared with cells treated with solvent control, 1 μmol/L ZLDI-8 still disrupted the activation of Notch pathway and thereby enhanced the effect of Sorafenib on MHCC97-H cells. This means that ZLDI-8 could enhance the sensitivity of HCC cells to anti-tumor agents with high safety capacity and potential application.

Notch family proteins are a series of transmembrane proteins. In response to cell-stress, e.g., ionizing radiation or cytotoxic chemotherapeutic agents, Notch proteins can be cleaved and activated by ADMA17, a member of metalloproteinase family (step one cleaving), and a presenilin-dependent gamma secretase complex (step two cleaving)^55–57^. As a result, the NICD (Intracellular domain of Notch) is released and translocates into nucleus to mediate the transcription of downstream gene, e.g., pro-survival or EMT genes which is related to MDR or metastasis of human cancers. Therefore, ADAM-17 plays essential roles in Notch pathway transduction and targeting ADAM-17 would be a novel strategy for inhibiting of Notch activation^58–60^. In the present work, we identified ZLDI-8, a novel inhibitor of ADAM-17 and found that pre-treatment of ZLDI-8 enhanced the anti-tumor effect of Sorafenib and traditional chemotherapeutic agents via in vitro or in vivo models. Treatment of ZLDI-8 could inhibit the activation of ADAM-17, and disrupts the accumulation of NICD in HCC cells, especially in the nucleus. ZLDI-8 treatment also decreased the expression of pro-survival and anti-apoptosis regulators and inhibited the EMT process of HCC cells. This work provided the evidence that ZLDI-8 can be a novel sensitizer that make tumor cells susceptible to anti-tumor agents and therefore overcoming HCC MDR process.

## Electronic supplementary material

Supplementary figure legends

Supplemental Figure 1

Supplemental Figure 2

Supplemental Figure 3

Supplemental Figure 4

Supplement table
