# Supplementary material for: Novel ADAM-17 inhibitor ZLDI-8 enhances the in vitro and in vivo chemotherapeutic effects of Sorafenib on hepatocellular carcinoma cells
Source: Cell Death Dis. 2018 Jul 3;9(7):743. doi: 10.1038/s41419-018-0804-6 (PMC6030059; doi:10.1038/s41419-018-0804-6)
Supplement: Supplementary file 1 — Supplementary figure legends [file 41419_2018_804_MOESM1_ESM.doc]

**Supplemental Figure 1. The chemical structure of ZLDI-8.**

**Supplemental Figure 2. The effect of indicated concentration of ZLDI-8 on MHCC97-H cells’ subcutaneous growth.** MHCC97-H cells were seeded into nude mice to form subcutaneous tumors. (A-C) The anti-tumor activity of indicated concentration of ZLDI-8 were measured in xenograft nude mice. The results were shown as photographs (A), tumor volumes (B) or tumor weight (C). *p < 0.05 versus ZLDI-8 or control.

**Supplemental Figure 3. The effect of indicated concentration of ZLDI-8 on MHCC97-H cells’ subcutaneous growth.** MHCC97-H cells were seeded into nude mice to form subcutaneous tumors. (A-C) The anti-tumor activity of indicated concentration of ZLDI-8 were measured in xenograft nude mice. Next, the protein level of Ki67, a proliferation indicator of human tumor cells, in tumor tissues was examined by western blot. *p < 0.05 versus ZLDI-8 or control.

**Supplemental Figure 4. The Diagram of Notch pathway activation and ZLDI-8 function in this work.** (A) Notch was initially activated undergoing a cleavage by ADAM-17 and releasing the Notch Intracellular Domain (NICD). Next, NICD translocates to the nucleus and mediates the transcription of Notch pathway. (B) Notch pathway could be inhibited by ADAM-17 inhibitor, ZLDI-8.
