# Supplementary figures and images for: Novel ADAM-17 inhibitor ZLDI-8 enhances the in vitro and in vivo chemotherapeutic effects of Sorafenib on hepatocellular carcinoma cells
Source: Cell Death Dis. 2018 Jul 3;9(7):743. doi: 10.1038/s41419-018-0804-6 (PMC6030059; doi:10.1038/s41419-018-0804-6)

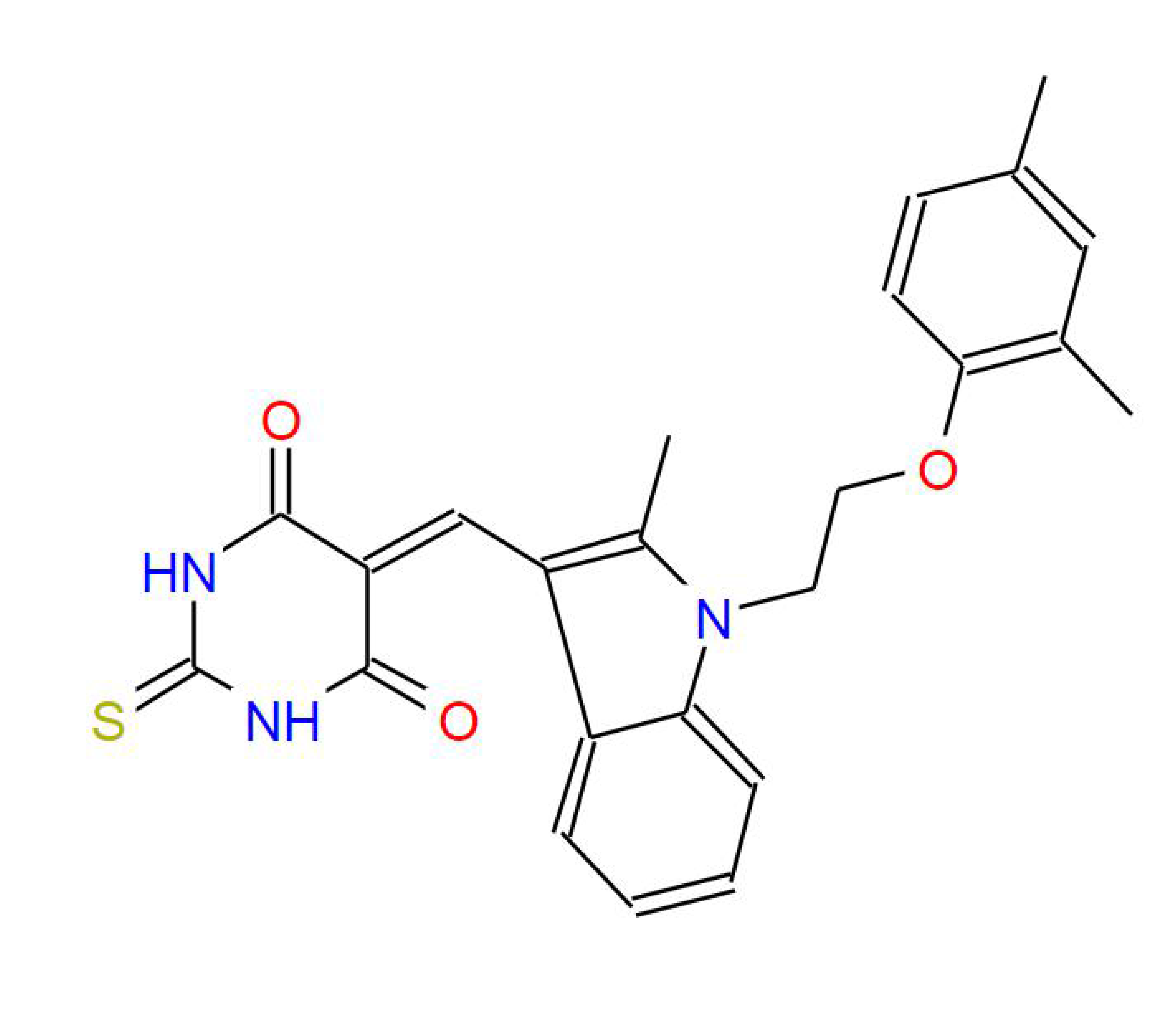

Supplement: Supplementary file 2 — Supplemental Figure 1 [file 41419_2018_804_MOESM2_ESM.tif]

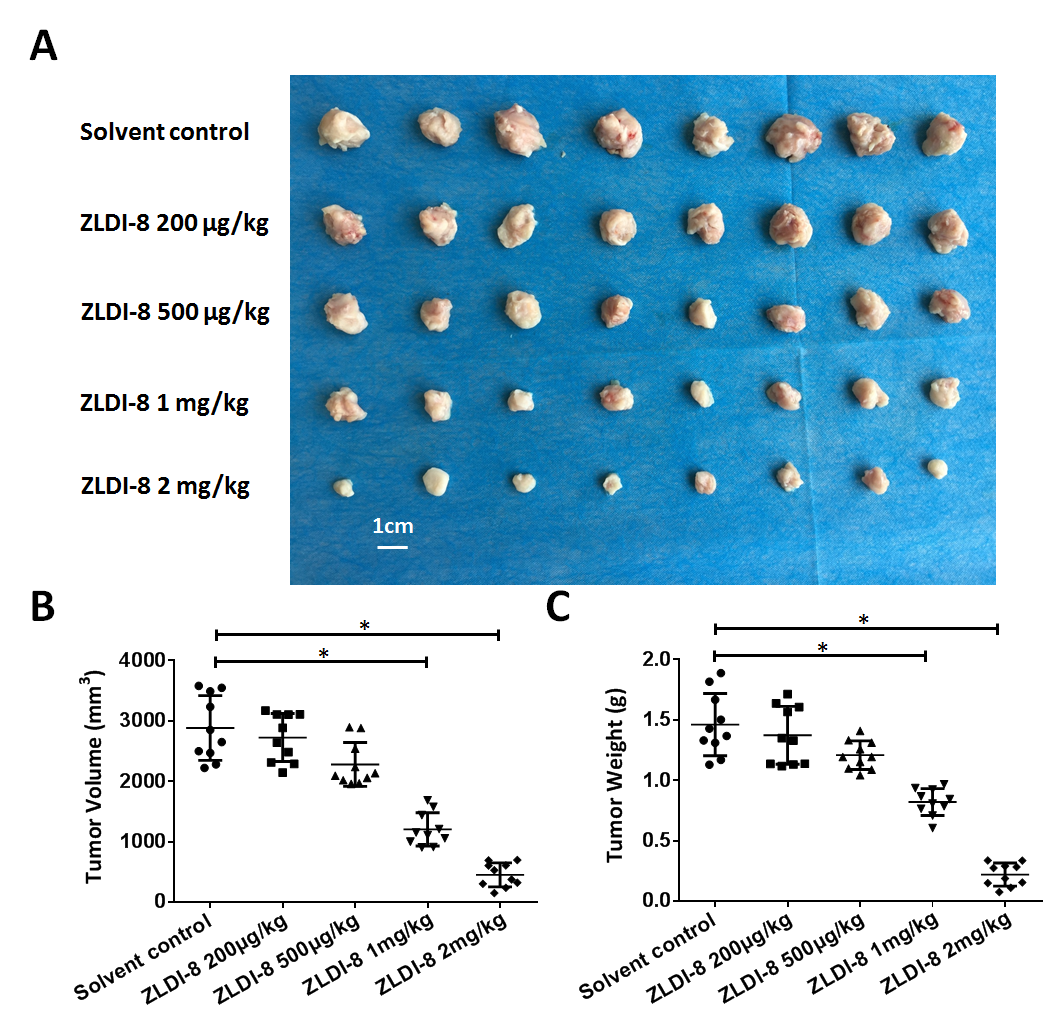

Supplement: Supplementary file 3 — Supplemental Figure 2 [file 41419_2018_804_MOESM3_ESM.tif]

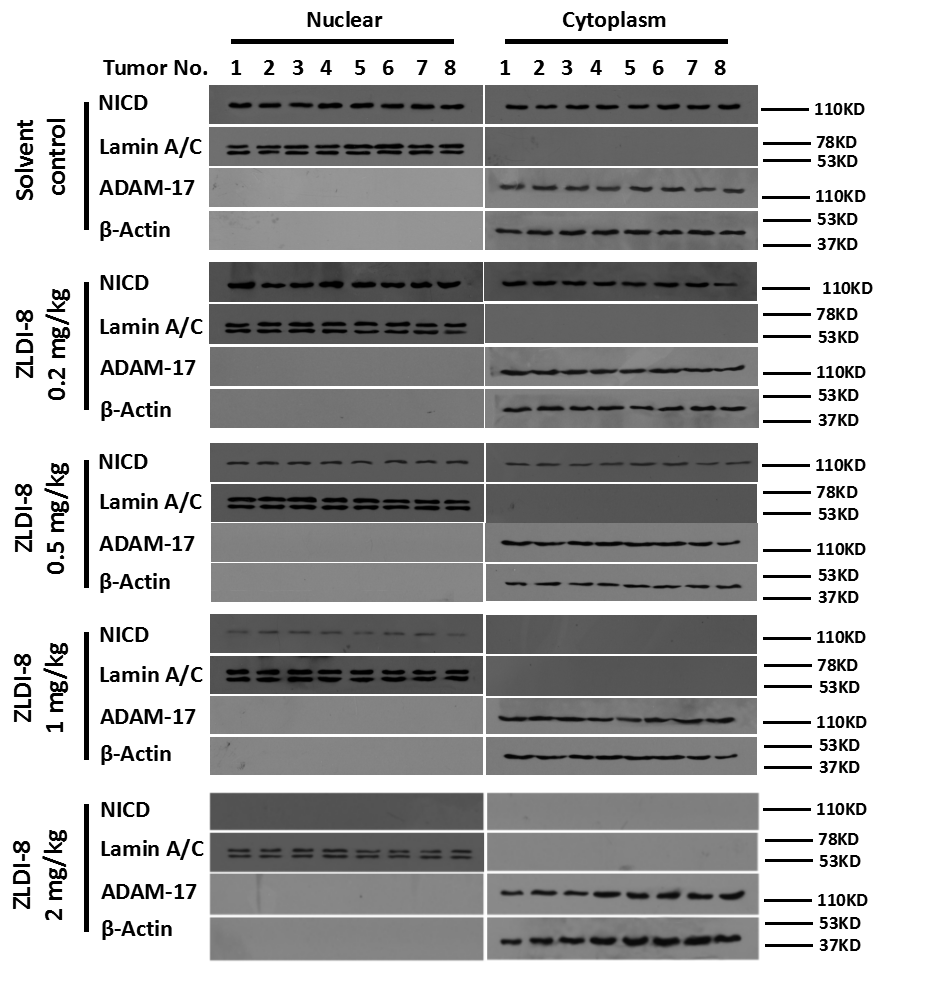

Supplement: Supplementary file 4 — Supplemental Figure 3 [file 41419_2018_804_MOESM4_ESM.tif]

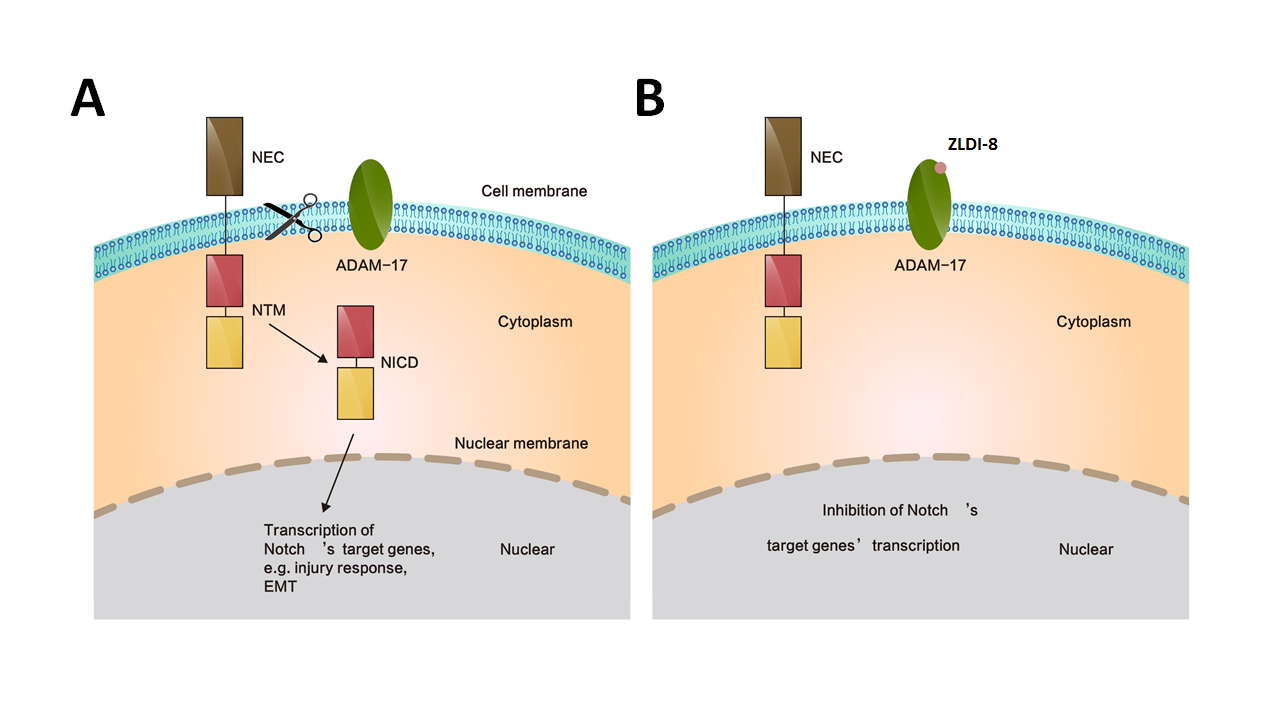

Supplement: Supplementary file 5 — Supplemental Figure 4 [file 41419_2018_804_MOESM5_ESM.tif]
