# Supplementary material for: Novel ADAM-17 inhibitor ZLDI-8 enhances the in vitro and in vivo chemotherapeutic effects of Sorafenib on hepatocellular carcinoma cells
Source: Cell Death Dis. 2018 Jul 3;9(7):743. doi: 10.1038/s41419-018-0804-6 (PMC6030059; doi:10.1038/s41419-018-0804-6)
Supplement: Supplementary file 6 — Supplement table [file 41419_2018_804_MOESM6_ESM.doc]

**Table S1. Concentrations of compounds used in this work.**

| **Compounds** | **Concentration (μmol/L)** | | | | | | |
| --- | --- | --- | --- | --- | --- | --- | --- |
| **IAC-8** | 0.030 | 0.100 | 0.300 | 1.000 | 3.000 | 10.00 | 30.00 |
| **Sorafenib** | 0.010 | 0.030 | 0.100 | 0.300 | 1.000 | 3.000 | 10.00 |
| **Etoposide** | 0.003 | 0.010 | 0.030 | 0.100 | 0.300 | 1.000 | 3.000 |
| **Paclitaxel** | 0.001 | 0.003 | 0.010 | 0.030 | 0.100 | 0.300 | 1.000 |
